# Salicylic Acid and Mobile Regulators of Systemic Immunity in Plants: Transport and Metabolism

**DOI:** 10.3390/plants12051013

**Published:** 2023-02-23

**Authors:** Tae-Jin Kim, Gah-Hyun Lim

**Affiliations:** 1 Department of Integrated Biological Science, College of Natural Sciences, Pusan National University, Busan 46241, Republic of Korea; 2 Department of Biological Sciences, College of Natural Sciences, Pusan National University, Busan 46241, Republic of Korea; 3 Institute of Systems Biology, Pusan National University, Busan 46241, Republic of Korea

**Keywords:** azelaic acid, glycerol-3-phosphate, SA transport, salicylic acid, systemic acquired resistance

## Abstract

Systemic acquired resistance (SAR) occurs when primary infected leaves produce several SAR-inducing chemical or mobile signals that are transported to uninfected distal parts via apoplastic or symplastic compartments and activate systemic immunity. The transport route of many chemicals associated with SAR is unknown. Recently, it was demonstrated that pathogen-infected cells preferentially transport salicylic acid (SA) through the apoplasts to uninfected areas. The pH gradient and deprotonation of SA may lead to apoplastic accumulation of SA before it accumulates in the cytosol following pathogen infection. Additionally, SA mobility over a long distance is essential for SAR, and transpiration controls the partitioning of SA into apoplasts and cuticles. On the other hand, glycerol-3-phosphate (G3P) and azelaic acid (AzA) travel via the plasmodesmata (PD) channel in the symplastic route. In this review, we discuss the role of SA as a mobile signal and the regulation of SA transport in SAR.

## 1. Introduction

Salicylic acid (SA, 2-hydroxybenzoic acid) is an essential defense hormone in plants that accumulates upon a pathogen attack to induce local defense and systemic acquired resistance (SAR) [1,2]. Genetic analyses have revealed that genes related to SA biosynthesis, conjugation, accumulation, signaling, and crosstalk with other hormones have been characterized, providing insights into how the immune response is finely coordinated [3,4,5,6,7,8,9]. The effects of SA can change enzyme activity, increase defense genes, enhance several defense responses, and/or generate free radicals [10]. Moreover, the evidence that SA plays a role in signaling resistance has been corroborated by analyses of *Arabidopsis* and tobacco plants, which accumulate little or no SA due to the expression of the bacterial *nahG* gene, which encodes salicylate hydroxylase [11]. SA biosynthesis and its complex role in plant defense are not yet fully understood, despite extensive research over the past 30 years. Although numerous studies have shown that SA plays a crucial role in SAR, its specific mechanism remains relatively unexplored.

SAR provides long-lasting protection against secondary pathogen attacks by priming the plant’s defense response. SAR is triggered by an initial infection, and results in the activation of the plant’s defense response throughout the entire plant, even in tissues that are not yet infected. As a result of SAR, the plant is able to coordinate its defense response and respond appropriately to pathogen attacks, even in distant tissues. Although the identity of a specific mobile signal is unknown, numerous SAR-inducing chemicals have been identified, some of which are physically mobile, and some of which are volatile in nature. These include salicylic acid (SA) [12] and its derivative methyl SA (MeSA) [13], pipecolic acid (Pip) [14] and its derivative N-hydroxy Pip (NHP) [15], dehydroabietinal (DA) [16], free radicals, nitric oxide (NO), reactive oxygen species (ROS) [17], azelaic acid (AzA) [18], glycerol-3-phosphate (G3P) [19], pinene volatiles [20], and extracellular (e)NAD(P) [21]. Several proteins also play crucial roles in SAR, including cuticle formation proteins such as acyl carrier protein 4 (ACP4) and mosaic death 1 (MOD1) [22]; two plasmodesmata (PD)-located proteins, such as plasmodesmata localizing protein 1/5 (PDLP1/5) [23,24]; and lipid transfer proteins (LTPs), such as defective induced resistance 1 (DIR1) and AzA insensitive 1 (AZI1) [25,26]. In recent years, the key components of the SAR pathway have been explored, with the goal of gaining a deeper understanding of how they work. Many of these SAR inducers appear to work in parallel pathways, with nonlinear interactions between them [19]. There are two different ways in which chemicals associated with SAR can be transported: via the apoplastic compartments or via the symplastic compartments [23]. Infection with pathogens can result in increased levels of SA and G3P/AzA in the apoplastic and symplastic compartments, respectively [23]. Other SAR-associated chemicals remain unknown in terms of their transport routes. Moreover, SA is partitioned into cuticle wax in greater quantities in mutants with defective cuticles and increased transpiration, suggesting that transpiration pull may also play an important role in SA transport [27]. This review focuses on the role of SA in SAR, and discusses recent studies showing that intact cuticles are necessary for systemic SA transport. Additionally, SA is discussed in relation to SAR components, including other mobile signals or proteins [27].

## 2. Salicylic Acid and Systemic Acquired Resistance

### 2.1. SA Biosynthesis

In higher plants, SA is synthesized from chorismate through two distinct metabolic pathways, each with multiple steps: the isochorismate synthase (ICS)- and phenylalanine ammonia-lyase (PAL)-derived pathways [28,29,30]. Different plant species have different branches toward SA biosynthesis. Pathogen infections in soybeans lead to equal contributions from the ICS and PAL pathways to the total pool of synthesized SA [30]. In *Arabidopsis*, the majority of pathogen-induced SA is derived from the ICS branch by plastid-localized ICS1 (also known as SA deficient 2 (SID2)) [31]. In *Arabidopsis*, there are two isochorismate synthase genes, ICS1 and ICS2; however, only ICS1 is induced by pathogens. The *ics1* mutants showed 90–95% less SA accumulation after pathogen infection than wild-type plants [31]. Only the *ics1/ics2* double mutant strongly affected SA accumulation, indicating that ICS1 generates most of the SA via isochorismate during pathogen defense. [8,32]. Several *Arabidopsis* mutants with altered SA accumulation are known to result from mutations in three genes encoding ICS pathway components. According to recent studies, bacteria have the Isochorismate Pyruvate Lyase (IPL) enzyme, which directly converts isochorismate to SA, whereas *Arabidopsis* requires both AvrPphB-susceptible 3 (PBS3) and Enhanced Pseudomonas Susceptibility 1 (EPS1) [30,33,34,35,36,37]. The amidotransferase PBS3 catalyzes the conjugation of isochorismate to glutamate, resulting in the formation of isochorismate-9-glutamate (IC-9-Glu). Subsequently, either IC-9-Glu can spontaneously decay into SA or convert into SA by EPS1. PBS3 and EPS1 are located in the cytosol, whereas ICS is localized in the chloroplasts [30]. Accordingly, the transport of isochorismate from plastids to the cytosol is essential for SA production. EDS5 (Enhanced Disease Susceptibility 5) was originally believed to act as a transporter of SA on the chloroplast envelope when pathogen-induced SA accumulation occurred [38,39]. However, in the *eds5-3* mutants, co-expression of *ICS1* and engineered chloroplast-targeted PBS3 can restore SA biosynthesis, suggesting that EDS5 could participate in isochorismate transport to the cytosol during SA biosynthesis [30].

**Figure 1:**
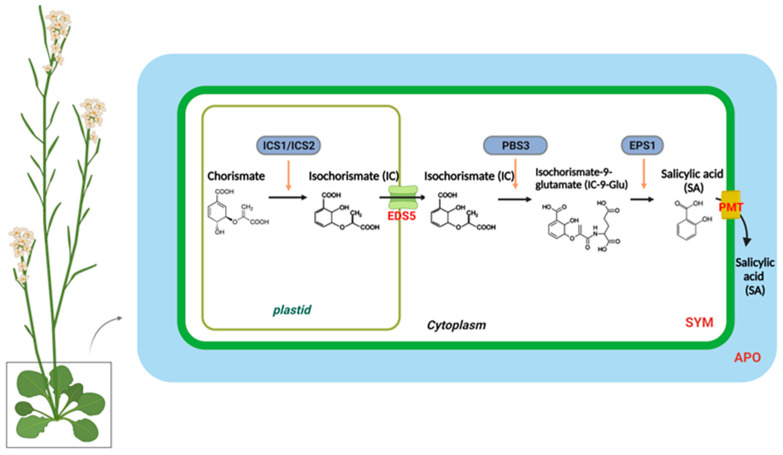
The biosynthesis and transport of salicylic acid in a plant cell. In plastids, chorismite is converted to isochorismate (IC) through the action of an IsoChorismate Synthase (ICS1/2). A MATE transporter called Enhanced Disease Susceptibility 5 (EDS5) transports IC from the plastid to the cytosol. The AvrPphB susceptible 3 (PBS3) catalyzes the conjugation of IC to glutamate (Glu), which results in IsoChorismate-9-Glutamate (IC-9-Glu). IC-9-Glu is then converted to salicylic acid (SA) by Enhanced Pseudomonas Susceptibility 1 (EPS1). SA moves preferentially to the apoplastic compartment (APO) through the plasma membrane transporter (PMT).

A small amount of SA accumulation is induced by pathogens in the *ics1/ics2* double mutant, which is blocked by the ICS pathway [33]. *Arabidopsis* contains four PAL genes, and studies with mutants or PAL-specific inhibitors suggest that the PAL pathway is also involved in SA biosynthesis [6,40]. Nevertheless, the PAL quadruple mutants still have approximately 25% of the wild-type basal SA levels and approximately 50% of the induced SA levels following pathogen infection [40]. The PAL pathway involves trans-cinnamic acid synthesized from phenylalanine, which is then converted to SA via benzoic acid [41] (Figure 1).

### 2.2. Regulation of SA Biosynthesis

The expression of *ICS1* is rapidly induced upon pathogen infection, which dramatically increases the SA levels. In recent reviews [2,42], a large number of transcriptional and post-transcriptional regulators were identified that affect *ICS1*. Furthermore, *EDS5* and *PBS3* were strongly induced during infection. These three SA biosynthesis genes, as well as a number of immune regulator genes, are controlled by the transcription factors SAR-Deficient 1 (*SARD1*) and Calmodulin-Binding Protein 60-Like.g (CBP60g) [43]. The transcription of SARD1 and CBP60g is stimulated by TGACG-Binding Factors1 (TGA1) and TGA4, respectively, whereas the negative immune regulators Calmodulin-Binding Transcription Factor 1 (CAMTA1), CAMTA2, and CAMT3 inhibit their transcription by inhibiting expression [44]. In addition, a pathogen induces the expression of *ICS1, EDS5,* and *PBS3* by binding to its promoter with CBP60g, and is homologous to SARD1 [45]. The *cbp60g sard1* double mutant impairs *ICS1*, *EDS5*, and *PBS3* induction and, subsequently, SA biosynthesis by bacterial elicitor flg22 or the avirulent bacterial strain *Pseudomonas syringae* pv *maculicola (Psm)* ES4326 avrB, resulting in compromised pattern-triggered immunity (PTI)- and effector-triggered immunity (ETI)-induced resistance as well as SAR [46,47]. Mutants defective in *EDS5* show impaired SA accumulation and resistance to *Pseudomonas syringae* pv. tomato (*Pst*) DC3000 [8]. Likewise, *pbs3* mutants display reduced SA accumulation in response to pathogens, such as plants with defects in ICS1 and EDS5. Additionally, several proteins contribute to SA accumulation and SAR in response to pathogens. These include Enhanced Disease Susceptibility 1 (*EDS1*), Phytoalexin Deficient 4 (*PAD4*), and Non-race-specific Disease Resistance 1 (*NDR1*), which show partial reductions in SA levels, unlike ICS1 and EDS5 [48,49,50,51,52,53,54,55,56,57,58,59]. 

### 2.3. SA Signaling Components

The SA signaling pathway in *Arabidopsis* thaliana is activated by two receptor classes: Nonexpressor of Pathogenesis-Related Genes (NPR1) and NPR1-Like Protein 3/4 (NPR3/4). This stimulates the expression of defense-related genes and immunity. Neither *NPR1* nor *NPR3/NPR4* contain DNA-binding domains; they must interact with transcription factors, such as TGA2/5/6, for signal transduction [60,61,62,63,64]. NPR1 was identified through genetic analysis of the SA-insensitive mutants. NPR1 is a transcriptional coactivator essential for the expression of pathogenesis-related (PR) genes, as well as broad-spectrum disease resistance. Unlike NPR1, NPR3 and NPR4 negatively regulate plant defense, with *npr3/npr4* mutants showing enhanced PR gene expression and basal resistance. NPR3 and NPR4 were identified as SA receptors based on their high SA affinities. NPR4 has an SA-binding core domain that refolds with SA to form a helical fold that completely encloses SA within its hydrophobic core. The interaction between NPR1 and NPR4 is disrupted by SA-induced conformational changes in the core of NPR4, which binds to SA. SA binding by NPR1 has also been reported, although its activity varies between studies. Similarly to their negative association with SA signaling, NPR3 and NPR4 are CRL3 substrate receptors for NPR1 polyubiquitination and degradation. SA-mediated gene expression is controlled by NPR3 and NPR4 by controlling NPR1 stability [65]. A negative regulator of SA signaling, *NIM1-Interacting 1 (NIMIN1)*, is strongly induced by SA on top of NPR3 and NPR4 [66]. NIMIN1 negatively regulates NPR1-mediated immune responses by interacting with NPR1. The *nimin1* mutant resulted in enhanced SA-induced *PR1* expression, while *NIMIN1* overexpression exhibited phenotypes similar to *npr1* mutants, including reduced *PR1* expression after SA induction, compromised SAR, and SA intolerance [66,67]. SAR is dependent upon the activation of NPR1 and the inhibition of NPR3/NPR4 by SA, both of which contribute to PTI and ETI [66]. 

### 2.4. Regulation of SA Transport in SAR

Several studies have shown that SA is crucial for SAR, but its role has been largely unclear until recently. Mobile inducers of SAR are produced in the primary infected leaves, and are then translocated to distal uninfected portions to activate SAR [68,69,70,71,72]. In addition, numerous SAR-inducing chemicals have been identified, conferring systemic resistance when applied exogenously, and play a crucial role in pathogen-induced immunity. Among these chemicals, SA, which is essential for SAR, accumulates in infected and uninfected tissues to a lesser extent [4]. Exogenous SA or its analogs, such as 1,2,3-benzothiadiazole-7-carbothioic acid and S-methyl ester (BTH), can induce SAR without infection [73]. In plants, the phloem can be loaded via apoplastic or symplastic routes. The apoplast is the space between the plasma membrane and cell wall, while the symplast is a network of cytoplasms connected by the plasmodesmata. AzA and G3P are transported from the local to distal tissues via the symplastic route, whereas SA is transported by the extracytosolic apoplastic compartment [23]. In early research on SAR, SA was not considered a mobile signal because it was likely to be synthesized de novo in uninfected leaves rather than mobilized from the infection sites. These results are based on graft studies performed on transgenic tobacco plants expressing a bacterial salicylate hydroxylase (NahG) that converts SA into catechol. SAR was induced in wild-type scions as a result of primary infection in nahG rootstocks, whereas primary infection in wild-type rootstocks was unable to induce SAR in nahG scions [11]. Vernooij et al. [11] suggested that an additional, as of yet unknown, signal induces SA synthesis in distal tissues. These results indicate that SA is not transported to distal tissues, but rather, it already exists in these tissues and is required for SAR activation [11]. However, recent studies have shown the systemic mobility of SA in wild-type and nahG plants [27]. Furthermore, pathogen infection increases SA levels in the apoplastic compartment, and SA is exported to the apoplast before accumulating in the cytosol [27]. Since SA has a low pKa value (2.98), the COOH group is mainly deprotonated (COO-) at a neutral to slightly alkaline pH (7–7.5) of the cytosol. To avoid rapid cytosolic pH increases caused by protons released by accumulating SA, pathogen-induced SA may be exported to the apoplast based on the pH and the presence of known proton pump inhibitors sodium orthovanadate and omeprazole [27]. Thus, it is likely that proton pumps play a role in SA transport, since both chemicals inhibited SA transport in a concentration-dependent manner [27]. This suggests that cytosolic nahG might be unable to access apoplastic SA, which could be easily transported into the phloem and moved systemically. In fact, a recent study showed systemic SA transport in *Arabidopsis* and tobacco nahG plants, although the levels were lower than in wild-type plants [27]. Additionally, transport of ^14^C-SA to distal tissues was confirmed after infiltration in local leaves in wild-type and nahG *Arabidopsis* plants.

### 2.5. Regulatory Role of the Cuticle in SA Transport

Intriguingly, active SA transport is regulated by the plant cuticle. An intact cuticle is also required for systemic SA transport, since a portion of total SA is partitioned into cuticle wax. Mutants with defects in their cuticles, such as the *acp4* and *mod1* mutants, which exhibit increased transpiration, tend to have higher levels of SA in their cuticle waxes, suggesting that the transpiration pull regulates its partitioning [23]. For example, mutations in *ACP4*, a component of fatty acid (FA) biosynthesis and lipid synthesis initiation, can reduce wax components (FA, alkanes, and primary alcohols) and cutin aliphatic monomers [22]. Moreover, mutations in *MOD1* result in a defective enoyl-ACP reductase enzyme that is necessary for the production of FA, resulting in reduced levels of multiple FA species as well as total lipids. Interestingly, in both *acp4* and *mod1* mutant plants, the transport of SA into petiole exudate (PEX) and apoplasts was impaired, even though the levels of SA in the infected leaves was similar to that in wild-type leaves [22]. A previous study showed that cuticle defects were associated with compromised SAR, but no explanation was given for how cuticles contributed to compromised SAR [22]. Recently, evidence has shown that *acp4* and *mod1* mutants had lower water usage efficiency levels (WUE), and did not transport SA efficiently through the apoplast. As a consequence, both *acp4* and *mod1* mutant plants had low water potential and increased stomatal aperture. These results indicate that increased transpiration decreases apoplastic hydrostatic pressure in these mutants, which allows SA to enter the cuticle [27,74,75]. This correlates with the fact that high relative humidity growth conditions can reduce water loss in *acp4* and *mod1* mutants and restore systemic SA transport and SAR. Interestingly, *sid2* mutants exhibited reduced water potential, reduced WUE, and increased stomatal aperture. In addition, exogenous SA restored stomatal aperture and water potential in these plants, but not pathogen infection. In contrast, following both exogenous SA and pathogen infection, the *acp4* and *mod1* mutant plants had reduced stomatal apertures. SA treatment did not induce jasmonic acid (JA) levels or abscisic acid (ABA) levels, which suggests that SA is independent of ABA. Therefore, the function of SA and ABA in relation to stomatal aperture needs to be further investigated. Thus, the balance between cuticular SA levels and intracellular water potential can regulate stomatal opening [76].

## 3. Mobile Inducers of SAR

### 3.1. Biosynthesis of G3P and AzA in SAR

Pathogen infections result in rapid NO accumulation during SAR via unknown mechanisms. NO application did not confer SAR to *Respiratory Burst Oxidase Homologs (RBOH)* mutants rbohD and rbohF, indicating that ROS radicals function downstream of NO in SAR [21]. As a result of pathogen infection, these mutants do not accumulate superoxide radicals. There is no redundancy between *rbohD* and *rbohF,* and mutation in either of them compromises SAR. Nevertheless, exogenous ROS application restored SAR in NO associated protein 1 (*noa1*)/nitrate reductases 1 (*nia1*) or the *noa1/nia2* double mutants, which were deficient in NO [77]. Plants infected with pathogens accumulate AzA up to six-fold more in petiole exudates, and at least some of this AzA translocates to the distal tissues (up to 7%) [18,26]. A pathogen infection releases free FAs from membrane lipids that are hydrolyzed by ROS to generate AzA. Plant lipids digalactosyldiacylglycerol (DGDG) and monogalatosyldiacylglycerol (MGDG) contain C18 FAs that are catalyzed by different ROS species [78]. In particular, AzA is formed by cleaving the double bonds between carbons 9 and 10 of C18 unsaturated FAs, including oleic (18:1), linoleic (18:2), and linolenic acids (18:3) [24]. G3P is a phosphorylated metabolite of glycerol that plays a crucial role in lipid metabolism. The biosynthesis of G3P in plants takes place mainly during photosynthesis. Some of the glucose produced is then converted into G3P through a series of metabolic reactions. G3P is synthesized from dihydroxyacetone phosphate (DHAP) by triose phosphate isomerase (TPI) [19]. Exogenous AzA increased the expression of the G3P biosynthesis genes *GLY1* (G3P dehydrogenase, G3PDH) and *GLI1* (glycerol kinase, GK), resulting in G3P accumulation [19]. Exogenous G3P causes resistance to *Colletotrichum higginsianum* and induces SAR in *Arabidopsis* and monocots [19,79]. Interestingly, plants do not accumulate SA following exogenous G3P and AzA administration, which induce SAR in wild-type plants. Despite this, neither G3P nor AzA is able to confer SAR in *ics1(sid2)* mutant plants, which have significantly reduced either basal or pathogen-induced SA levels [19]. As a result, SA is essential for establishing SAR, but the accumulation of SA alone is not sufficient. A defect in G3P synthesis in *gly1* and *gli1* mutant plants resulted in a compromised SAR phenotype, which could be restored by exogenous G3P application [19,26]. Intriguingly, *gly1* and *gli1* mutants exhibited levels of SA and AzA similar to those in the wild-type plants. Additionally, exogenous application of G3P alone was not sufficient to induce SA biosynthesis or SAR. These results suggest that, similarly to SA, AzA and G3P may not be sufficient to induce SAR.

### 3.2. Regulation of G3P and AzA Transport in SAR

**Figure 2:**
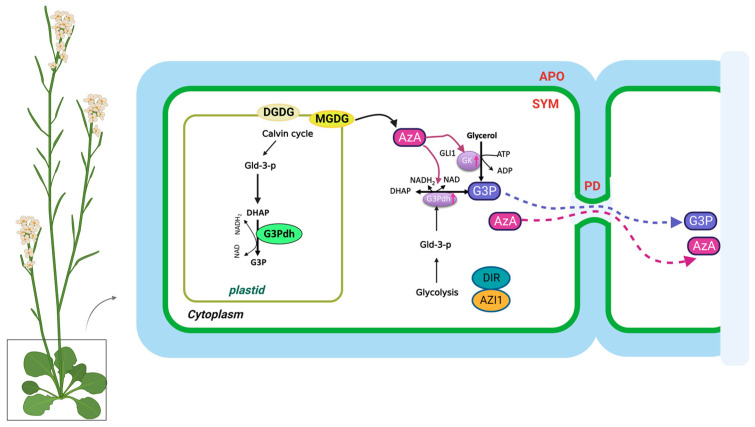
The biosynthesis and transport of azelaic acid and glycerol-3-phosphate in the plant cell. AzA and G3P are transported via the symplast through plasmodesmata (PD). AZA induces G3P biosynthesis through its effects on genes involved in SAR. DIR1 and AZI1, both of which depend on G3P for stability, are required for G3P-mediated SAR signaling. G3P is either converted to glycerol by G3P phosphatase or used to synthesize membrane lipids (glycerolipids) and triacylglycerol (TAG). The source of DHAP is glyceraldehyde-3-phosphate (Gld-3-P), which originates from glycolysis and the Calvin cycle. DGDG, galactolipid digalactosyldiacylglycerol; MGDG, monogalatosyldiacylglycerol; AzA, azelaic acid; G3P, glycerol-3-phosphate; Gld-3-P, Glyceraldehyde-3-Phosphate; DHAP, DHA phosphate; GK, glycerol kinase; G3Pdh, G3P dehydrogenase; DIR1, Defective Induced Resistance 1; AZI1, AzA Insensitive 1 (AZI1).

In the presence of DIR1, G3P is systemically mobile, but its direct binding has not yet been identified [26]. DIR1 is likely to be directly associated with this bioactive G3P-derivative, and upon translocation to distal tissues, induces SAR in response to pathogen infection. SAR does not seem to be associated with AzA transport, which may be explained by its independence from DIR1, AZI1, GLY1, GLI1, or exogenous G3P [26]. In contrast to SA, G3P and AzA are preferentially transported from local to distal leaves through the PD, and defects in PD permeability affect their transport (Figure 2). Thus, PD-localizing proteins (PDLPs) not only regulate PD permeability, thereby controlling AzA and G3P transport, but also signal through the SAR [23]. Plants overexpressing *PDLP5* have impaired AzA and G3P transport, as well as compromised SAR. In addition, 35S-PDLP5-expressing plants show reduced transport of DIR1, a lipid-transfer protein involved in SAR [23,24]. The lipid transfer-like protein AZI1, an important component of the SAR pathway, is also regulated by *PDLP1* and *PDLP5* [23]. Thus, SAR was impaired in both *pdlp1* and *pdlp5* mutant plants, but only pdlp5 plants showed an increase in PD permeability. As AZI1 interacts with both PDLP1 and PDLP5, it is likely to form complexes with both proteins. Loss of PDLP1 or PDLP5 increases the chloroplastic localization of AZI1, although the biological significance of the chloroplastic localization of AZI1 or its intracellular partitioning during SAR is not known [23,69]. Therefore, understanding how AzA functions in subcellular compartments is crucial to understanding SAR. G3P and AzA, which induce SAR in wild-type plants, do not induce SA accumulation in plants. In spite of this, neither G3P nor AzA can confer SAR in *ics1(sid2)* mutant plants, which have significantly reduced either basal or pathogen-induced SA levels.

### 3.3. Pip and NHP-Mediated SAR

The inoculation of pathogens results in massive metabolic changes in *Arabidopsis* and other plants which increase the formation of aromatic amino acids, branched-chain amino acids, and lysine [80]. A recently discovered plant metabolite, N-hydroxy-pipecolic acid (NHP), plays a vital role in SAR and is synthesized from lysine. In *Arabidopsis*, pipecolic acid (Pip) is converted into NHP in *Arabidopsis* by three different enzymes that respond strongly to biotic stresses [80]. Lysine is converted into 2,3-dehydro-pipecolic acid (dehydro-Pip; 2,3-DP) by the AGD2-like defense response protein 1 (ALD1), which encodes a Lys aminotransferase [80]. Then, 2,3-DP is converted into Pip by SAR-Deficient 4 (SARD4), which encodes the bacterial ornithine cyclodeaminase [80]. The final step is conversion of Pip into NHP by adding hydroxyl amines through flavin-dependent monooxygenase 1 (FMO1) [81,82]. Exogenous Pip also induces defense priming and increases the expression of genes associated with plant defenses, increasing local resistance to *P. syringae* [14,81,83]. *FMO1* overexpression conferred resistance to infection by bacterial and oomycete pathogens, whereas defects in *SARD4*, another Pip biosynthetic gene, eliminated resistance [80,81,82]. Wang et al. [84] detected localized application of C^14^-Pip in distal leaves and, similarly, Návarová et al. [14] detected Pip in vascular exudates after local infection. However, petiole exudate from Pip-deficient ald1 plants induces SAR, suggesting that Pip and NHP mobility might not be functionally necessary for SAR induction in the distal tissue [84,85]. Pathogen-infected plants accumulate NHP, and exogenous application of NHP restores SAR in *ald1* and *fmo1* mutants, suggesting that NHP may act downstream of Pip [80,81,82]. This evidence suggests that NHPs function as novel plant defense hormones that play a critical role in triggering systemic defense responses and SAR [80,81,82]. 

### 3.4. SA and NHP-Mediated SAR

Interestingly, NHP treatment significantly induces the expression of *NPR1* and *NPR3/NPR4*, suggesting that NHP may be involved in both regulating SA signaling output and promoting SA biosynthesis [81]. Considering the overlap between SA and NHP regulators, it is not surprising that SA and NHP can act in conjunction to induce SAR [86]. In addition, both NHP and SA are glycosylated by UDT76B1, a UDP-dependent glycosylase [80]. It has been shown that the loss-of-function mutation in UGT76B1 increases the levels of NHP and SA, and therefore enhances its resistance to pathogens. As a result of overexpression of *UGT76B1*, free NHP and SA levels are reduced, and the response to SARs is diminished [80]. Recent ChIP analyses revealed that *SARD1* and *CBP60g* target not only SA biosynthesis genes, but also genes involved in NHP biosynthesis [43]. Although the induction of *SARD1* and *CBP60g* during SAR is mediated by NHP, how NHP activates *SARD1* and *CBP60g* transcription remains unelucidated. In addition, NADP and NHP induce SA marker gene expression, but the specificity of the response is unclear. It remains unclear how SA and NHP biosynthesis is regulated by various transcription factors upstream of the defense signal pathway. It is necessary to conduct more research to understand how SA and NHP activate SAR.

## 4. Conclusions and Future Directions

Recent research has shown that the cuticle regulates SA transport during SAR, and SAR-inducing chemicals can be transported by either apoplastic or symplastic compartments. After pathogen infection, SA levels increase in the apoplastic compartment, suggesting that SA movement into the apoplastic compartment is critical to SAR. The partitioning of SA in the cuticle wax is increased in mutants with defective cuticles, which exhibit increased transpiration. Moreover, defective SA transport in defective cuticle mutants were due to significantly reduced SA levels in their PEX and apoplastic compartments after pathogen infection. This suggests that transpiration pull plays an important role in SA transport. However, the mechanisms involved in SAR and the role of chemical or mobile signals that induce SAR in plants remain unclear. The understanding of how SAR works can lead to a better understanding of how plants interact with their environments, leading to an improved understanding of plant disease management. SAR can be applied in agriculture for disease management by inducing resistance in crops in order to protect against pests and diseases. Furthermore, recent studies indicate that SA improves plants’ tolerance to abiotic stress by influencing several biochemical and physiological changes [87]. As an example, methyl salicylate (MeSA) was proposed as a signal molecule in plant responses to abiotic stresses because MeSA improved cucumber plants’ tolerance to chilling injury when soaked in seed [88]. Exogenous salicylic acid also increases polyamine content in maize, but it may reduce drought tolerance [89]. SAR research can also take on new directions, such as examining the role of the microbiome in SAR and assessing the potential of combining SAR with multiple biotic and abiotic factors. 

## Data Availability

Not applicable.

## References

[1] Vlot A.C., Dempsey D.M.A., Klessig D.F. (2009). Salicylic acid, a multifaceted hormone to combat disease. Annu. Rev. Phytopathol..

[2] Zhang Y., Li X. (2019). Salicylic acid: Biosynthesis, perception, and contributions to plant immunity. Curr. Opin. Plant Biol..

[3] Dempsey D.M.A., Vlot A.C., Wildermuth M.C., Klessig D.F. (2011). Salicylic acid biosynthesis and metabolism. Arab. Book/Am. Soc. Plant Biol..

[4] Gaffney T., Friedrich L., Vernooij B., Negrotto D., Nye G., Uknes S., Ward E., Kessmann H., Ryals J. (1993). Requirement of salicylic acid for the induction of systemic acquired resistance. Science.

[5] Delaney T.P., Uknes S., Vernooij B., Friedrich L., Weymann K., Negrotto D., Gaffney T., Gut-Rella M., Kessmann H., Ward E. (1994). A central role of salicylic acid in plant disease resistance. Science.

[6] Mauch-Mani B., Slusarenko A.J. (1996). Production of salicylic acid precursors is a major function of phenylalanine ammonia-lyase in the resistance of *Arabidopsis* to *Peronospora parasitica*. Plant Cell.

[7] Pallas J.A., Paiva N.L., Lamb C., Dixon R.A. (1996). Tobacco plants epigenetically suppressed in phenylalanine ammonia-lyase expression do not develop systemic acquired resistance in response to infection by tobacco mosaic virus. Plant J..

[8] Nawrath C., Métraux J.-P. (1999). Salicylic acid induction–deficient mutants of Arabidopsis express PR-2 and PR-5 and accumulate high levels of camalexin after pathogen inoculation. Plant Cell.

[9] Dewdney J., Reuber T.L., Wildermuth M.C., Devoto A., Cui J., Stutius L.M., Drummond E.P., Ausubel F.M. (2000). Three unique mutants of Arabidopsis identify eds loci required for limiting growth of a biotrophic fungal pathogen. Plant J..

[10] Dempsey D.M.A., Klessig D.F. (2012). SOS–too many signals for systemic acquired resistance?. Trends Plant Sci..

[11] Vernooij B., Friedrich L., Morse A., Reist R., Kolditz-Jawhar R., Ward E., Uknes S., Kessmann H., Ryals J. (1994). Salicylic acid is not the translocated signal responsible for inducing systemic acquired resistance but is required in signal transduction. Plant Cell.

[12] Shah J., Chaturvedi R., Chowdhury Z., Venables B., Petros R.A. (2014). Signaling by small metabolites in systemic acquired resistance. Plant J..

[13] Park S.-W., Kaimoyo E., Kumar D., Mosher S., Klessig D.F. (2007). Methyl salicylate is a critical mobile signal for plant systemic acquired resistance. Science.

[14] Návarová H., Bernsdorff F., Döring A.-C., Zeier J. (2012). Pipecolic acid, an endogenous mediator of defense amplification and priming, is a critical regulator of inducible plant immunity. Plant Cell.

[15] Hartmann M., Zeier J. (2018). l-lysine metabolism to N-hydroxypipecolic acid: An integral immune-activating pathway in plants. Plant J..

[16] Chaturvedi R., Venables B., Petros R.A., Nalam V., Li M., Wang X., Takemoto L.J., Shah J. (2012). An abietane diterpenoid is a potent activator of systemic acquired resistance. Plant J..

[17] Wang C., El-Shetehy M., Shine M., Yu K., Navarre D., Wendehenne D., Kachroo A., Kachroo P. (2014). Free radicals mediate systemic acquired resistance. Cell Rep..

[18] Jung H.W., Tschaplinski T.J., Wang L., Glazebrook J., Greenberg J.T. (2009). Priming in systemic plant immunity. Science.

[19] Chanda B., Xia Y., Mandal M.K., Yu K., Sekine K.T., Gao Q.-m., Selote D., Hu Y., Stromberg A., Navarre D. (2011). Glycerol-3-phosphate is a critical mobile inducer of systemic immunity in plants. Nat. Genet..

[20] Riedlmeier M., Ghirardo A., Wenig M., Knappe C., Koch K., Georgii E., Dey S., Parker J.E., Schnitzler J.-P., Vlot A.C. (2017). Monoterpenes support systemic acquired resistance within and between plants. Plant Cell.

[21] Sagi M., Fluhr R. (2006). Production of reactive oxygen species by plant NADPH oxidases. Plant Physiol..

[22] Xia Y., Gao Q.-M., Yu K., Lapchyk L., Navarre D., Hildebrand D., Kachroo A., Kachroo P. (2009). An intact cuticle in distal tissues is essential for the induction of systemic acquired resistance in plants. Cell Host Microbe.

[23] Lim G.-H., Shine M., de Lorenzo L., Yu K., Cui W., Navarre D., Hunt A.G., Lee J.-Y., Kachroo A., Kachroo P. (2016). Plasmodesmata localizing proteins regulate transport and signaling during systemic acquired immunity in plants. Cell Host Microbe.

[24] Carella P., Isaacs M., Cameron R. (2015). Plasmodesmata-located protein overexpression negatively impacts the manifestation of systemic acquired resistance and the long-distance movement of Defective in Induced Resistance1 in A rabidopsis. Plant Biol..

[25] Maldonado A.M., Doerner P., Dixon R.A., Lamb C.J., Cameron R.K. (2002). A putative lipid transfer protein involved in systemic resistance signalling in Arabidopsis. Nature.

[26] Yu K., Soares J.M., Mandal M.K., Wang C., Chanda B., Gifford A.N., Fowler J.S., Navarre D., Kachroo A., Kachroo P. (2013). A feedback regulatory loop between G3P and lipid transfer proteins DIR1 and AZI1 mediates azelaic-acid-induced systemic immunity. Cell Rep..

[27] Lim G.-H., Liu H., Yu K., Liu R., Shine M., Fernandez J., Burch-Smith T., Mobley J.K., McLetchie N., Kachroo A. (2020). The plant cuticle regulates apoplastic transport of salicylic acid during systemic acquired resistance. Sci. Adv..

[28] Huang W., Wang Y., Li X., Zhang Y. (2020). Biosynthesis and regulation of salicylic acid and N-hydroxypipecolic acid in plant immunity. Mol. Plant.

[29] Wildermuth M.C., Dewdney J., Wu G., Ausubel F.M. (2001). Isochorismate synthase is required to synthesize salicylic acid for plant defence. Nature.

[30] Rekhter D., Lüdke D., Ding Y., Feussner K., Zienkiewicz K., Lipka V., Wiermer M., Zhang Y., Feussner I. (2019). Isochorismate-derived biosynthesis of the plant stress hormone salicylic acid. Science.

[31] Gao Q.-m., Kachroo A., Kachroo P. (2014). Chemical inducers of systemic immunity in plants. J. Exp. Bot..

[32] Garcion C., Lohmann A., Lamodière E., Catinot J., Buchala A., Doermann P., Métraux J.-P. (2008). Characterization and biological function of the ISOCHORISMATE SYNTHASE2 gene of Arabidopsis. Plant Physiol..

[33] Mercado-Blanco J.S., van der Drift K.M., Olsson P.E., Thomas-Oates J.E., van Loon L.C., Bakker P.A. (2001). Analysis of the pmsCEAB gene cluster involved in biosynthesis of salicylic acid and the siderophore pseudomonine in the biocontrol strain Pseudomonas fluorescens WCS374. J. Bacteriol..

[34] Jagadeeswaran G., Raina S., Acharya B.R., Maqbool S.B., Mosher S.L., Appel H.M., Schultz J.C., Klessig D.F., Raina R. (2007). Arabidopsis GH3-LIKE DEFENSE GENE 1 is required for accumulation of salicylic acid, activation of defense responses and resistance to Pseudomonas syringae. Plant J..

[35] Lee M.W., Lu H., Jung H.W., Greenberg J.T. (2007). A key role for the Arabidopsis WIN3 protein in disease resistance triggered by Pseudomonas syringae that secrete AvrRpt2. Mol. Plant Microbe Interact..

[36] Nobuta K., Okrent R., Stoutemyer M., Rodibaugh N., Kempema L., Wildermuth M., Innes R. (2007). The GH3 acyl adenylase family member PBS3 regulates salicylic acid-dependent defense responses in Arabidopsis. Plant Physiol..

[37] Torrens-Spence M.P., Bobokalonova A., Carballo V., Glinkerman C.M., Pluskal T., Shen A., Weng J.-K. (2019). PBS3 and EPS1 complete salicylic acid biosynthesis from isochorismate in Arabidopsis. Mol. Plant.

[38] Nawrath C., Heck S., Parinthawong N., Métraux J.-P. (2002). EDS5, an essential component of salicylic acid–dependent signaling for disease resistance in Arabidopsis, is a member of the MATE transporter family. Plant Cell.

[39] Serrano M., Wang B., Aryal B., Garcion C., Abou-Mansour E., Heck S., Geisler M., Mauch F., Nawrath C., Métraux J.-P. (2013). Export of salicylic acid from the chloroplast requires the multidrug and toxin extrusion-like transporter EDS5. Plant Physiol..

[40] Huang J., Gu M., Lai Z., Fan B., Shi K., Zhou Y.-H., Yu J.-Q., Chen Z. (2010). Functional analysis of the Arabidopsis PAL gene family in plant growth, development, and response to environmental stress. Plant Physiol..

[41] Yalpani N., León J., Lawton M.A., Raskin I. (1993). Pathway of salicylic acid biosynthesis in healthy and virus-inoculated tobacco. Plant Physiol..

[42] Peng Y., Yang J., Li X., Zhang Y. (2021). Salicylic acid: Biosynthesis and signaling. Annu. Rev. Plant Biol..

[43] Sun T., Zhang Y., Li Y., Zhang Q., Ding Y., Zhang Y. (2015). ChIP-seq reveals broad roles of SARD1 and CBP60g in regulating plant immunity. Nat. Commun..

[44] Sun T., Huang J., Xu Y., Verma V., Jing B., Sun Y., Orduna A.R., Tian H., Huang X., Xia S. (2020). Redundant CAMTA transcription factors negatively regulate the biosynthesis of salicylic acid and N-hydroxypipecolic acid by modulating the expression of *SARD1* and *CBP60g*. Mol. Plant.

[45] Truman W., Glazebrook J. (2012). Co-expression analysis identifies putative targets for CBP60g and SARD1 regulation. BMC Plant Biol..

[46] Wang L., Tsuda K., Truman W., Sato M., Nguyen L.V., Katagiri F., Glazebrook J. (2011). CBP60g and SARD1 play partially redundant critical roles in salicylic acid signaling. Plant J..

[47] Zhang Y., Xu S., Ding P., Wang D., Cheng Y.T., He J., Gao M., Xu F., Li Y., Zhu Z. (2010). Control of salicylic acid synthesis and systemic acquired resistance by two members of a plant-specific family of transcription factors. Proc. Natl. Acad. Sci. USA.

[48] Century K.S., Holub E.B., Staskawicz B.J. (1995). NDR1, a locus of Arabidopsis thaliana that is required for disease resistance to both a bacterial and a fungal pathogen. Proc. Natl. Acad. Sci. USA.

[49] Falk A., Feys B.J., Frost L.N., Jones J.D., Daniels M.J., Parker J.E. (1999). EDS1, an essential component of R gene-mediated disease resistance in Arabidopsis has homology to eukaryotic lipases. Proc. Natl. Acad. Sci. USA.

[50] Jirage D., Tootle T.L., Reuber T.L., Frost L.N., Feys B.J., Parker J.E., Ausubel F.M., Glazebrook J. (1999). Arabidopsis thaliana PAD4 encodes a lipase-like gene that is important for salicylic acid signaling. Proc. Natl. Acad. Sci. USA.

[51] McDowell J.M., Dangl J.L. (2000). Signal transduction in the plant immune response. Trends Biochem. Sci..

[52] Feys B.J., Moisan L.J., Newman M.-A., Parker J.E. (2001). Direct interaction between the Arabidopsis disease resistance signaling proteins, EDS1 and PAD4. EMBO J..

[53] Coppinger P., Repetti P.P., Day B., Dahlbeck D., Mehlert A., Staskawicz B.J. (2004). Overexpression of the plasma membrane-localized NDR1 protein results in enhanced bacterial disease resistance in Arabidopsis thaliana. Plant J..

[54] Ishihara T., Sekine K.T., Hase S., Kanayama Y., Seo S., Ohashi Y., Kusano T., Shibata D., Shah J., Takahashi H. (2008). Overexpression of the Arabidopsis thaliana EDS5 gene enhances resistance to viruses. Plant Biol..

[55] Bhattacharjee S., Halane M.K., Kim S.H., Gassmann W. (2011). Pathogen effectors target Arabidopsis EDS1 and alter its interactions with immune regulators. Science.

[56] Cacas J.-L., Petitot A.-S., Bernier L., Estevan J., Conejero G., Mongrand S., Fernandez D. (2011). Identification and characterization of the Non-race specific Disease Resistance 1 (NDR1) orthologous protein in coffee. BMC Plant Biol..

[57] Heidrich K., Wirthmueller L., Tasset C., Pouzet C., Deslandes L., Parker J.E. (2011). Arabidopsis EDS1 connects pathogen effector recognition to cell compartment–specific immune responses. Science.

[58] Knepper C., Savory E.A., Day B. (2011). Arabidopsis NDR1 is an integrin-like protein with a role in fluid loss and plasma membrane-cell wall adhesion. Plant Physiol..

[59] Zhang P.-J., Li W.-D., Huang F., Zhang J.-M., Xu F.-C., Lu Y.-B. (2013). Feeding by whiteflies suppresses downstream jasmonic acid signaling by eliciting salicylic acid signaling. J. Chem. Ecol..

[60] Liu Y., Sun T., Sun Y., Zhang Y., Radojičić A., Ding Y., Tian H., Huang X., Lan J., Chen S. (2020). Diverse roles of the salicylic acid receptors NPR1 and NPR3/NPR4 in plant immunity. Plant Cell.

[61] Zhang Y., Fan W., Kinkema M., Li X., Dong X. (1999). Interaction of NPR1 with basic leucine zipper protein transcription factors that bind sequences required for salicylic acid induction of the PR-1 gene. Proc. Natl. Acad. Sci. USA.

[62] Després C., DeLong C., Glaze S., Liu E., Fobert P.R. (2000). The Arabidopsis NPR1/NIM1 protein enhances the DNA binding activity of a subgroup of the TGA family of bZIP transcription factors. Plant Cell.

[63] Zhou J.-M., Trifa Y., Silva H., Pontier D., Lam E., Shah J., Klessig D.F. (2000). NPR1 differentially interacts with members of the TGA/OBF family of transcription factors that bind an element of the PR-1 gene required for induction by salicylic acid. Mol. Plant Microbe Interact..

[64] Zhang J.-P., Chen Q.-X., Song K.-K., Xie J.-J. (2006). Inhibitory effects of salicylic acid family compounds on the diphenolase activity of mushroom tyrosinase. Food Chem..

[65] Fu Z.Q., Yan S., Saleh A., Wang W., Ruble J., Oka N., Mohan R., Spoel S.H., Tada Y., Zheng N. (2012). NPR3 and NPR4 are receptors for the immune signal salicylic acid in plants. Nature.

[66] Weigel R.R., Pfitzner U.M., Gatz C. (2005). Interaction of NIMIN1 with NPR1 modulates PR gene expression in Arabidopsis. Plant Cell.

[67] Mohan R., Tai T., Chen A., Arnoff T., Fu Z.-Q. (2016). Overexpression of Arabidopsis NIMIN1 results in salicylate intolerance. Plant Signal. Behav..

[68] Gao Q.-M., Zhu S., Kachroo P., Kachroo A. (2015). Signal regulators of systemic acquired resistance. Front. Plant Sci..

[69] Wendehenne D., Gao Q.-m., Kachroo A., Kachroo P. (2014). Free radical-mediated systemic immunity in plants. Curr. Opin. Plant Biol..

[70] Singh A., Lim G.H., Kachroo P. (2017). Transport of chemical signals in systemic acquired resistance. J. Integr. Plant Biol..

[71] Shine M., Xiao X., Kachroo P., Kachroo A. (2019). Signaling mechanisms underlying systemic acquired resistance to microbial pathogens. Plant Sci..

[72] Rasmussen J.B., Hammerschmidt R., Zook M.N. (1991). Systemic induction of salicylic acid accumulation in cucumber after inoculation with Pseudomonas syringae pv syringae. Plant Physiol..

[73] Yalpani N., Silverman P., Wilson T., Kleier D.A., Raskin I. (1991). Salicylic acid is a systemic signal and an inducer of pathogenesis-related proteins in virus-infected tobacco. Plant Cell.

[74] Kachroo P., Liu H., Kachroo A. (2020). Salicylic acid: Transport and long-distance immune signaling. Curr. Opin. Virol..

[75] Kachroo A., Liu H., Yuan X., Kurokawa T., Kachroo P. (2022). Systemic acquired resistance-associated transport and metabolic regulation of salicylic acid and glycerol-3-phosphate. Essays Biochem..

[76] Buckley T.N. (2019). How do stomata respond to water status?. New Phytol..

[77] El-Shetehy M., Wang C., Shine M., Yu K., Kachroo A., Kachroo P. (2015). Nitric oxide and reactive oxygen species are required for systemic acquired resistance in plants. Plant Signal. Behav..

[78] Gao Q.-m., Yu K., Xia Y., Shine M., Wang C., Navarre D., Kachroo A., Kachroo P. (2014). Mono-and digalactosyldiacylglycerol lipids function nonredundantly to regulate systemic acquired resistance in plants. Cell Rep..

[79] Yang Y., Zhao J., Liu P., Xing H., Li C., Wei G., Kang Z. (2013). Glycerol-3-phosphate metabolism in wheat contributes to systemic acquired resistance against *Puccinia striiformis* f. sp. *tritici*. PLoS ONE.

[80] Zeier J. (2021). Metabolic regulation of systemic acquired resistance. Curr. Opin. Plant Biol..

[81] Hartmann H., Moura C.F., Anderegg W.R., Ruehr N.K., Salmon Y., Allen C.D., Arndt S.K., Breshears D.D., Davi H., Galbraith D. (2018). Research frontiers for improving our understanding of drought-induced tree and forest mortality. New Phytol..

[82] Chen Y.-C., Holmes E.C., Rajniak J., Kim J.-G., Tang S., Fischer C.R., Mudgett M.B., Sattely E.S. (2018). N-hydroxy-pipecolic acid is a mobile metabolite that induces systemic disease resistance in *Arabidopsis*. Proc. Natl. Acad. Sci. USA.

[83] Bernsdorff F., Döring A.-C., Gruner K., Schuck S., Bräutigam A., Zeier J. (2016). Pipecolic acid orchestrates plant systemic acquired resistance and defense priming via salicylic acid-dependent and-independent pathways. Plant Cell.

[84] Wang C., Liu R., Lim G.-H., de Lorenzo L., Yu K., Zhang K., Hunt A.G., Kachroo A., Kachroo P. (2018). Pipecolic acid confers systemic immunity by regulating free radicals. Sci. Adv..

[85] Shine M., Zhang K., Liu H., Lim G.-h., Xia F., Yu K., Hunt A.G., Kachroo A., Kachroo P. (2022). Phased small RNA–mediated systemic signaling in plants. Sci. Adv..

[86] Shields A., Shivnauth V., Castroverde C.D.M. (2022). Salicylic Acid and N-Hydroxypipecolic Acid at the Fulcrum of the Plant Immunity-Growth Equilibrium. Front. Plant Sci..

[87] Khan M.I.R., Fatma M., Per T.S., Anjum N.A., Khan N.A. (2015). Salicylic acid-induced abiotic stress tolerance and underlying mechanisms in plants. Front. Plant Sci..

[88] Gondor O.K., Pál M., Janda T., Szalai G. (2022). The role of methyl salicylate in plant growth under stress conditions. J. Plant Physiol..

[89] Nemeth M., Janda T., Horvath E., Paldi E., Szalai G. (2002). Exogenous salicylic acid increases polyamine content but may decrease drought tolerance in maize. Plant Sci..

